# Changes in self-harm- and violence-related urgent psychiatric consultation in the emergency department during the different stages of the COVID-19 pandemic

**DOI:** 10.1186/s12888-022-04029-4

**Published:** 2022-06-07

**Authors:** Chun-Hao Liu, Po-Cheng Chen, Jian-Hong Chen, Chung-Cheng Yeh

**Affiliations:** 1 Department of Child Psychiatry, Chang Gung Memorial Hospital at Linkou, Taoyuan city, Taiwan; 2 College of Medicine, Chang Gung University, Taoyuan city, Taiwan; 3 Department of Rehabilitation Medicine, Kaohsiung Chang Gung Memorial Hospital, Kaohsiung city, Taiwan; 4 Department of Psychiatry, Chang Gung Memorial Hospital at Keelung, Keelung city, Taiwan; 5 National Taiwan Sport University, Taoyuan city, Taiwan; 6 Department of Emergency Medicine, Chang Gung Memorial Hospital at Keelung, Keelung city, Taiwan

**Keywords:** COVID-19, emergency department, psychiatric consultation, self-harm, violence

## Abstract

**Background:**

The coronavirus disease 2019 pandemic significantly affected emergency department (ED) visits and urgent psychiatric consultation (UPC) seeking behavior in EDs. Our study explored the changes in UPCs during and after the pandemic peak.

**Methods:**

This retrospective observational study evaluated UPCs in the ED of a referral medical center in Taiwan, where treated both physical and psychiatric complaints. We defined the COVID-19 pandemic peak period as calendar week 4–18, 2020. The corresponding baseline as calendar week 4–18, 2019, and the slack period as week 4–18, 2021. The total number of UPCs, patient demographic data such as sex and age of the patients seen, the referral system (whether police or emergency medical service [EMS] or other sources), and the chief complaint (self-harm or violence) were recorded.

**Results:**

Compared with the baseline period, a significant decline in UPCs was observed in the pandemic peak period, and a rebound was observed in the slack period, with the median [IQR] Q1, Q3 values of 22 [18, 26], 12 [10, 17]), and 16 [15, 23], respectively. We observed significantly few men (34.9% vs 45.2%) and less violence (10.2% vs 17.6%) in the peak period compared with in the baseline period, but no significant difference was found compared with the slack period. Throughout the pandemic, younger patients (41.8 ± 17.4 in 2019, 39.2 ± 18.5 [*p* = 0.121] in 2020, and 35.6 ± 17.2 [*p* < 0.001] in 2021), higher proportions of police/EMS referral (38.7% in 2019, 41.9% [*p* = 0.473] in 2020, and 51.9% [*p* = 0.001] in 2021) and self-harm–related complaints (57% in 2019, 62.4% [*p* = 0.233] in 2020, and 64.9% [*p* = 0.049] in 2021) was noted among UPC seekers during the pandemic. However, the proportion of violence-related UPCs (17.6% in 2019, 10.2% [*p* = 0.023] in 2020, and 12.3% [*p* = 0.072] in 2021) declined.

**Conclusions:**

This study found that UPCs changed throughout the pandemic. This result raises the concern that mental health needs are masked during the pandemic.

**Supplementary Information:**

The online version contains supplementary material available at 10.1186/s12888-022-04029-4.

## Background

The coronavirus disease 2019 (COVID-19) pandemic prominently changed people’s daily lives and health-seeking behaviors. In the United States, daily visits to emergency departments (EDs) of all causes decreased by approximately 50% across different states [1]. This trend of ED visit reduction during the epidemic was observed in Taiwan in 2003, where the severe acute respiratory syndrome epidemic caused a 51.6% reduction in ED visits [2]. ED visits decreased significantly during the COVID-19 epidemic peak in Taiwan [3]. Many possible factors might have contributed to decreased ED visits, but one hypothesis is that people would have avoided ED visits because of the fear of being infected by the COVID-19 virus [4].

The COVID-19 pandemic can affect people’s mental health in many aspects. A recent review suggested that this effect is contributed to by psychological, social and populational, and biological factors [5]. However, the fear of COVID-19 may keep people with mental health problems from seeking help from an ED. Studies have revealed an approximately 30% decrease in psychiatric ED visits during the pandemic [6, 7]. Furthermore, the characteristics of people visiting psychiatric EDs have changed. Compared with the baseline period of 2018–2019, a high proportion of patients with anxiety disorders, personality disorders, psychosis, and posttraumatic stress disorders visited EDs in 2020, while a decreased proportion of adjustment disorder was noted at the same time [7]. This phenomenon indicated that people who sought psychiatric consultation in EDs during the COVID-19 peak period may be different from those in the pre-COVID-19 era.

In Taiwan, two COVID-19 epidemic peaks occurred locally. The first one was from January 20, 2020, when the first case of COVID-19 was detected, to June 7, 2020, when local restrictions eased [8]. The second epidemic peak was from May 19, 2021, to July 26, 2021, and was a national level-3 epidemic [8]. However, unlike in most other countries, a slack period occurred between the two peaks, when people in Taiwan could have relatively normal lives. This feature may help us to know how the pandemic affected patients who visited ED for psychiatric help. The aim of our study was to demonstrate the change in the number of urgent psychiatric consultations (UPCs) during the peak and slack periods of the COVID-19 epidemic and demographic characteristics and suicide/violence-related complaints among people who received UPC in different epidemic periods.

## Methods

### Study design

In this retrospective observational study, data of psychiatric consultation were collected from the ED of a referral medical center in Northern Taiwan, where treating both physical and psychiatric complaints. During the pandemic, the ED remained open for psychiatric emergencies for walk-in or police/emergency medical service (EMS) referral patients. The study was approved by the Institutional Review Board of Chang Gung Memorial Hospital (No. 202101591B0).

### Definition of pandemic stages

We defined calendar week 4–18, 2020 as our study periods of the COVID-19 pandemic as our previous study [3]. This period began from the first case of COVID-19 in Taiwan a two weeks spare from the 2021 peak period to avoid overlap [3]. As its counterparts, calendar week 4–18 of 2019 and 2021 were defined as the baseline and slack periods, respectively.

### Data collection

All UPCs initiated by ED physicians during the study period were recorded. All the patients of UPCs were attended by resident psychiatrists who made the consultation notes. Furthermore, the total number of UPCs, demographic data of patients, referral source (through police/EMS or not), and presentation of self-harm or violence as the chief complaint were obtained from medical records. We included nonsuicidal self-injury, suicide ideation, and suicide attempt in the “self-harm” category because it was difficult to differentiate these chief complaints in the ED. Similarly, we included interpersonal violence and aggression toward objects in the “violence” category. Medical records encrypted in the electrical administration system due to legal reasons or other causes were excluded.

### Statistical analysis

To reduce the effect of extreme values, we used the median (interquartile range, IQR) to present ED visit and UPCs in each week. After testing normality by using the Shapiro–Wilk test, each week’s ED visits and UPCs were compared with the corresponding data in the baseline period by using the Mann–Whitney test. Each factor of the UPC is presented using number and percentage. All the data of the peak (2020) and slack (2021) periods were compared with those of the baseline period (2019). In the same period, the gender difference and the demographic data of patients who sought UPCs with and without self-harm or violence were compared. The Pearson chi-square test was used to analyze categorical variables such as sex and police/EMS referrals, and the independent t-test was used for analyzing continuous variables such as age. We defined statistical significance as two-tailed *p* < 0.05.

## Results

During the study period (week 4–18) of each year, total 324 UPCs were recorded in 2019 (baseline period), which then decreased to 190 UPCs in 2020 (peak period) and rebounded to 271 in 2021 (slack period). In the medical records, one, three, and three records were missing in 2019, 2020, and 2021, respectively, and one medical record in 2020 was encrypted. In total 323 (22 [18, 26] per week presented as median [IQR]), 186 (12 [10, 17] per week), and 268 (16 [15, 23] per week) UPCs were enrolled in our study in 2019, 2020, and 2021, respectively (Table 1). Most patients who sought UPC already had a recordable previous psychiatric history, while the number and percentage were 240 (74.3%), 129 (69.4%), and 145 (78%) in 2019, 2020, and 2021, respectively. For reference, the median (IQR) of the total ED visits of each week, that is, 3319 [3207, 3422], 2007 [1794, 2455], and 2573 [2484, 2700] were recorded in 2019, 2020, and 2021, respectively.

**Table 1:** Comparison of the weekly ED visits and UPCs (presented by median [IQR]) during the different pandemic stages

	ED visit per week		UPC per week	
2019 (baseline)	3319 [3207, 3422]	Reference	22 [18, 26]	Reference
2020 (peak)	2007 [1794, 2455]	***p* < 0.001**	12 [10, 17]	***p* = 0.001**
2021 (slack)	2573 [2484, 2700]	***p* < 0.001**	16 [15, 23]	*p* = 0.088

### Demographic data of patients who received a UPC

In 2019, 45.2% of the UPCs were by men, which decreased to 34.9% (*p* = 0.024) in 2020 (peak period) and rebounded to 41% (*p* = 0.310) in 2021 (slack period; Table 2). During the pandemic, we found younger patients visited ED for UPCs compared to baseline. The average ages (mean ± standard deviation) of these patients in 2019, 2020, and 2021 were 41.8 ± 17.4 (from 12 to 100), 39.2 ± 18.5 (from 4 to 93, *p* = 0.121), and 35.6 ± 17.2 (from 3 to 90, *p* < 0.001), respectively.

**Table 2:** Demographic data, referral source, and self-harm or violence-related behaviors of patients who opted for an UPC during the different pandemic stages

	2019 (baseline)	2020 (peak)		2021 (slack)	
	*n* = 323	*n* = 186		*n* = 268	
Sex (Male)	146 (45.2%)	65 (34.9%)	***p* = 0.024**	110 (41%)	*p* = 0.310
Age (mean ± SD)	41.8 ± 17.4	39.2 ± 18.5	*p* = 0.121	35.6 ± 17.2	***p* < 0.001**
Police/EMS	125 (38.7%)	78 (41.9%)	*p* = 0.473	139 (51.9%)	***p* = 0.001**
Self-harm	184 (57%)	116 (62.4%)	*p* = 0.233	174 (64.9%)	***p* = 0.049**
Violence	57 (17.6%)	19 (10.2%)	***p* = 0.023**	33 (12.3%)	*p* = 0.072

Police/EMS referral and risky behaviors.

The percentage of police/EMS referral increased after the pandemic, with 38.7% in 2019, 41.9% in 2020 (*p* = 0.473), and 51.9% in 2021 (*p* = 0.001; Table 2). The proportion of patients who sought UPCs due to self-harm showed increasing during the pandemic, with 57% in 2019, 62.4% in 2020 (*p* = 0.233), and 64.9% in 2021 (*p* = 0.049). However, for violence case, it was 17.6% in 2019, 10.2% in 2020 (*p* = 0.023), and 12.3% in 2021 (*p* = 0.072).

### Change in the demographic data of UPCs due to self-harm or violence during the pandemic

Among patients who sought UPCs due to self-harm, the proportion of men was significantly low (27.6%, *p* = 0.016) in the peak period, but not in the slack period (36.2%, *p* = 0.323), compared with the baseline (41.3%; Table 3). Conversely, among patients who sought UPCs due to violence, the proportion of men was high during the baseline, peak, and slack periods of the pandemic (57.9%, 73.7%, and 72.7%, respectively), but without statistical significance. Younger patients (aged 40 ± 17, 36.4 ± 17.2, and 33.5 ± 16.5 years) and higher proportion of police/EMS referral (40.8%, 42.2%, and 56.3%) was observed throughout the pandemic, with significance in the slack period. However, this phenomenon was not observed in the violence group (Table 4).

**Table 3:** Demographic data of patients who opted for an UPC due to self-harm–related behaviors

	2019 (baseline)			2020 (peak)			2021 (slack)		
	No self-harm *n* = 139	Self-harm *n* = 184	*P* value	No self-harm *n* = 70	Self-harm *n* = 116	*P* value	No self-harm *n* = 94	Self-harm *n* = 174	*P* value
Sex (male)	70 (50.4%)	76 (41.3%)	***P* = 0.105**	33 (47.1%)	32 (27.6%)	***P* = 0.007**	47 (50%)	63 (36.2%)	***P* = 0.028**
		Reference			***P* = 0.016**			*P* = 0.323	
Age	44.2 ± 17.8	40 ± 17	***p* = 0.034**	43.9 ± 19.8	36.4 ± 17.2	***P* = 0.008**	39.7 ± 17.9	33.5 ± 16.5	***P* = 0.005**
		Reference			*P* = 0.08			***P* < 0.001**	
Police/EMS	91 (64.7%)	75 (40.8%)	***P* = 0.027**	29 (41.4%)	49 (42.2%)	*P* = 0.913	41 (43.6%)	98 (56.3%)	***P* = 0.047**
		Reference			*P* = 0.800			***P* = 0.003**

**Table 4:** Demographic data of patients who opted for an UPC due to violence-related behaviors

	2019 (baseline)			2020 (peak)			2021 (slack)		
	No violence *n* = 266	Violence *n* = 57	*P* value	No violence *n* = 167	Violence *n* = 19	*P* value	No Violence *n* = 235	Violence *n* = 33	*P* value
Sex (male)	113 (42.5%)	33 (57.9%)	***P* = 0.034**	51 (30.5%)	14 (73.7%)	***P* < 0.001**	86 (36.6%)	24 (72.7%)	***P* < 0.001**
		Reference			*P* = 0.220			*P* = 0.159	
Age	41.9 ± 17.5	41.4 ± 17.1	*p* = 0.858	38.3 ± 18.2	47.8 ± 19.5	***P* = 0.032**	35.4 ± 17.2	37.5 ± 17.9	*P* = 0.519
		Reference			*P* = 0.176			*P* = 0.301	
Police/EMS	93 (35%)	32 (56.1%)	***P* = 0.003**	66 (39.5%)	12 (63.2%)	***P* = 0.048**	115 (48.9%)	24 (72.7%)	***P* = 0.01**
		Reference			*P* = 0.592			*P* = 0.118	

### Gender difference on the UPC patterns

We found there was some significant differences between male and female on the pattern of UPCs during the pandemic. For male patients, there was a significantly higher proportion of police/EMS referral (for male and female, 45.2% vs 33.3%, 56.9% vs 33.9%, 60% vs 46.2% in 2019, 2020, and 2021 respectively) and violence (22.6% vs 13.6%, 21.5% vs 4.1%, and 21.8% vs 5.7%; Table 5). For female, there was a higher proportion of self-harm behavior (52.1% vs 61%, 49.2% vs 69.4%, and 57.3% vs 70.2%), with significance in 2020 and 2021. There were significant younger female patients sought UPCs (age 39.9 ± 17.5 years for male and 32.7 ± 16.4 for female, *p* = 0.001) during the slack period.

**Table 5:** The UPC patterns between male and female

	Age	Police/EMS	Self-harm	Violence
2019 (baseline)				
Male (*n* = 146)	41.9 ± 17.1	66 (45.2%)	76 (52.1%)	33 (22.6%)
Female (*n* = 177)	41.7 ± 17.9	59 (33.3%)	108 (61%)	24 (13.6%)
*p*-value	0.918	**0.029**	0.105	**0.034**
2020 (peak)				
Male (*n* = 65)	41.8 ± 17.7	37 (56.9%)	32 (49.2%)	14 (21.5%)
Female (*n* = 121)	37.9 ± 18.9	41 (33.9%)	84 (69.4%)	5 (4.1%)
*p*-value	0.174	**0.002**	**0.007**	** < 0.001**
2021 (slack)				
Male (*n* = 110)	39.9 ± 17.5	66 (60%)	63 (57.3%)	24 (21.8%)
Female (*n* = 158)	32.7 ± 16.4	73 (46.2%)	111 (70.2%)	9 (5.7%)
*p*-value	**0.001**	**0.026**	**0.028**	** < 0.001**

## Discussion

In our study, we found a significant reduction in the total ED visits and UPCs during the peak period of the local epidemic, followed by a rebound during the slack period. Patients who needed UPCs were mostly women, and few patients has chief complaints of violence-related events during the peak period. Moreover, the proportions of young patients, patients referred by police or EMS, and patients who sought UPCs for self-harm were high among those who sought UPCs throughout the pandemic, and these significantly increased in the slack period.

The effect of COVID-19 on suicidal behavior is crucial in establishing mental health policies and psychiatric care, particularly for policies on self-harm and suicide. A recent meta-analysis revealed that the event rates of suicide ideation, suicide attempt, and self-harm behavior were 10.81%, 4.68%, and 9.63%, respectively, during the COVID-19 pandemic. [9] In a meta-analysis article in the pre-pandemic era, suicide ideation was 5.8% for 1-year prevalence [10], while another review article found the pooled 1-year prevalence for suicide ideation and suicide attempt were 3.62% and 0.57% in the Europe. [11] A cross sectional study in England revealed that life-time prevalence of non-suicidal self-injury was 2.4% in 2000, then it rose to 6.4% in 2014. [12] Despite these studies cannot be compared directly because of different design and population, it still indicated that suicide ideation, suicide attempt, and self-harm behavior were higher during the pandemic.

The relationship between COVID-19 pandemic and suicidal behavior is complex. A review article summarized possible factors contributed to suicide during the pandemic, including social isolation, panic, uncertainty, unemployment, and immune-mediated mechanism such as the increasing of proinflammatory cytokines due to COVID-19. [13] Knowing how the pandemic affect suicidal behavior can provide us a biopsychosocial model to approach the complex nature of suicide. However, some studies have reported different results. Data from Israel and Denmark showed no significant change in the proportion of self-harm–related disorder in psychiatric visits during the pandemic [7, 14], while other studies showed a decreasing suicide rate during the pandemic, especially in early stage [15, 16]. This controversial result means the effect of the pandemic on suicidal behavior may be inconsistent among countries and need further investigation. Although the total number of self-harm–related ED visits was lower during the pandemic than before the pandemic, our study revealed an increasing proportion of patients with self-harm–related chief complaints in EDs and UPCs after the COVID-19 pandemic. This delay may have resulted from the fear of being infected by the virus, leading to avoidance of medical help–seeking behavior. Our result suggests that self-harm behavior may have been masked during the pandemic and became a crucial mental health concern after the pandemic subsided.

Among patients with behavioral problems related to self-harm, the proportions of patients who were younger and referred by police/EMS were high during the pandemic, and the proportion of female patients increased during the epidemic peak period. Women and younger age were frequently identified as a risk factor of mental distress during the pandemic [17, 18]. Furthermore, another survey revealed the prevalence of more female patients than male patients among young patients who visited EDs for suicide-related behaviors during the pandemic [19]. A US national surveillance of persons aged 12–25 years revealed a decrease in ED visits at the beginning of the pandemic, followed by an increase, particularly among women [20]. This phenomenon may have resulted from psychosocial factors, such as financial crisis, unemployment, or stress from family members, affecting young women, which might have led to self-harm behaviors during the pandemic. A national surveillance showed that 10.7% responders had severe suicide ideation in the past month, and the phenomenon was significantly more prominent in female patients, minority population, unpaid caregivers, and essential workers [21]. Furthermore, unemployment during the pandemic may be related to an increased suicide rate [22]. Although no clear evidence is available to determine psychosocial factors directly contributing to their distress, younger people and women may be the most vulnerable with low financial support during the pandemic because of the social disadvantage.

Most studies on ED visits for violence have focused on intimate partner violence (IPV) during the pandemic. IPV has multiple aspects, namely individual, relationship, and societal factors, whereas social isolation, economic crisis, and government service unavailability due to the pandemic may exacerbate the risk [23]. During the pandemic, a 24/7 close interaction with a partner with a violence tendency may precipitate the risk. Studies on the change of the domestic violence rate during the pandemic reported controversial results. [24–26]. This different may related to different countries and different methodology. Although we do not identify the violence type in the UPC, a significant decline was observed during the pandemic. A Canadian study found that ED visits related to sexual assault and domestic violence declined during the pandemic [27]. Similarly, a large-scale survey in the U.S. revealed a reduced number of ED visit due to IPV during the pandemic [28]. Our findings may be due a decreased violence rate, but it may also indicate that victims were not seeking medical help during the pandemic.

This study had several limitations. First, the data were obtained from a single medical center, and therefore, the results cannot be generalized. Although the study site is the only referral center for nearby two counties and two cities (the demographic data of the coverage area see supplementary table 1) and therefore is representative, multicenter or population-based studies are necessary in future to confirm our preliminary findings. Second, we did not include data of years earlier than 2019 as baseline, and therefore, some bias may have occurred based on difference between years. More data from preceding years is needed to understand the trend of UPCs. Third, our study did not explore data regarding UPCs in detail, and therefore, the cause and nature of the self-harm or violence behavior or the final diagnosis cannot be determined.

## Conclusion

Our study revealed that the proportion of young patients, patients seeking help for self-harm–related behavior, and patients with police/EMS referral were high throughout the pandemic and increased further during the slack period. This may raise the concern that mental health care needs are masked, particularly self-harm or violence, during the pandemic peak period.

## Supplementary Information

**Additional file 1:** **Supplementary Table 1.** The demographic data of the nearby counties and cities at the end of 2010. **Supplementary Table 2.** The type of self-harm behaviors before and during the pandemic. **Supplementary Table 3.** The type of violence before and during the pandemic.

**Additional file 2.**

## Data Availability

All data generated or analysed during this study are included in this published article and its supplementary information files.
