# Supplementary material for: Changes in self-harm- and violence-related urgent psychiatric consultation in the emergency department during the different stages of the COVID-19 pandemic
Source: BMC Psychiatry. 2022 Jun 7;22:384. doi: 10.1186/s12888-022-04029-4 (PMC9171742; doi:10.1186/s12888-022-04029-4)
Supplement: Supplementary file 1 — Additional file 1: Supplementary Table 1. The demographic data of the nearby counties and cities at the end of 2010. Supplementary Table 2. The type of self-harm behaviors before and during the pandemic. Supplementary Table 3. The type of violence before and during the pandemic. [file 12888_2022_4029_MOESM1_ESM.docx]

Supplementary table 1. The demographic data of the nearby counties and cities at the end of 2010.

|  | Population | Male | Female | M:F | Age |
| --- | --- | --- | --- | --- | --- |
| Taoyuan city | 1,107,819 | 1,107,819 | 1,082,523 | 102.1 | 35.4 |
| Hsinchu city | 476,273 | 237,266 | 239,007 | 102.4 | 35.2 |
| Hsinchu county | 522,163 | 266,179 | 255,984 | 107.4 | 35.4 |
| Miaoli county | 530,339 | 265,612 | 264,727 | 102.7 | 38.7 |

Reference:

Data from the latest general survey of 2010, Directorate - General of Budget, Accounting and Statistics, Executive Yuan, Taiwan. <https://mobile.stat.gov.tw/CheckBoxListTable.aspx?T=VC41LjMuMg==> (Accessed on 2022/05/19)

Supplementary table 2. The type of self-harm behaviors before and during the pandemic.

|  | 2019  n=184 | 2020  n=116 | 2021  N=174 |
| --- | --- | --- | --- |
| Cutting/stabbing | 30 (16.3%) | 11 (9.5%) | 44 (25.3%) |
| Drug overdose | 79 (42.9%) | 43 (37.1%) | 45 (25.9%) |
| Jumping | 8 (4.3%) | 7 (6%) | 14 (8%) |
| Charcoal burning | 8 (4.3%) | 4 (3.4%) | 7 (4%) |
| Pesticide | 3 (1.6%) | 1 (0.9%) | 3 (1.7%) |
| Alkaline or acid | 6 (3.3%) | 7 (6%) | 1 (0.6%) |
| Hanging | 2 (1.1%) | 2 (1.7%) | 1 (0.6%) |
| Other | 21 (11.4%) | 2 (1.7%) | 11 (6.3%) |
| Multiple methods | 11 (6%) | 8 (6.9%) | 16 (9.2%) |

Supplementary table 3. The type of violence before and during the pandemic.

|  | 2019  n=57 | 2020  n=19 | 2021  N=33 |
| --- | --- | --- | --- |
| To object | 15 (26.3%) | 3 (15.8%) | 12 (36.4%) |
| Interpersonal | 42 (73.7%) | 15 (78.9%) | 16 (48.5%) |
